# Decellularized extracellular matrix mediates tissue construction and regeneration

**DOI:** 10.1007/s11684-021-0900-3

**Published:** 2021-12-28

**Authors:** Chuanqi Liu, Ming Pei, Qingfeng Li, Yuanyuan Zhang

**Affiliations:** 1 Department of Plastic and Burn Surgery, West China Hospital, Sichuan University, Chengdu 610041, China;; 2 Department of Plastic and Reconstructive Surgery, Shanghai Ninth People’s Hospital, Shanghai Jiao Tong University School of Medicine, Shanghai 200011, China;; 3 Stem Cell and Tissue Engineering Laboratory, Department of Orthopaedics, West Virginia University, Morgantown, WV 26506, USA;; 4 Wake Forest Institute for Regenerative Medicine, Wake Forest University Health Sciences, Winston-Salem, NC 27109, USA

**Keywords:** decellularized extracellular matrix, 3D culture, organoids, tissue repair

## Abstract

Contributing to organ formation and tissue regeneration, extracellular matrix (ECM) constituents provide tissue with three-dimensional (3D) structural integrity and cellular-function regulation. Containing the crucial traits of the cellular microenvironment, ECM substitutes mediate cell–matrix interactions to prompt stem-cell proliferation and differentiation for 3D organoid construction *in vitro* or tissue regeneration *in vivo*. However, these ECMs are often applied generically and have yet to be extensively developed for specific cell types in 3D cultures. Cultured cells also produce rich ECM, particularly stromal cells. Cellular ECM improves 3D culture development *in vitro* and tissue remodeling during wound healing after implantation into the host as well. Gaining better insight into ECM derived from either tissue or cells that regulate 3D tissue reconstruction or organ regeneration helps us to select, produce, and implant the most suitable ECM and thus promote 3D organoid culture and tissue remodeling for *in vivo* regeneration. Overall, the decellularization methodologies and tissue/cell-derived ECM as scaffolds or cellular-growth supplements used in cell propagation and differentiation for 3D tissue culture *in vitro* are discussed. Moreover, current preclinical applications by which ECM components modulate the wound-healing process are reviewed.

## Introduction

As a three-dimensional (3D) network in biology, extracellular matrix (ECM) provides a microenvironment to cells for homeostasis, ingrowth, tissue formation, and repair [1]. Each tissue or organ has its own ECM with a distinct composition, which is generated in the early stages of embryonic development and constantly remodeled to control 3D tissue homeostasis [2]. Tissue-specific ECM offers optimal cell–cell and cell–ECM interactions by mimicking native signaling events [3]. Cell–ECM interactions are crucial for modulating cell behaviors, functions, and fates [4]. During tissue repair, quantitative and qualitative changes occur in ECM compounds during 3D tissue remodeling, which is regulated by specific enzymes, including matrix metalloproteinases (MMPs) [5].

The principle of cell-based bioengineering aims to (1) develop *in vitro* 3D culture models, such as organoid formation; and (2) regenerate damaged tissues and organs with a combination of cells and ECM scaffolds. Previous studies have reported the use of various synthetic scaffolds mimicking the 3D ECM for tissue regeneration. For example, the pLOXL1-Lipo@PLCL-HA co-delivery system reportedly promotes pelvic-floor repair in rabbits [6], and 3D electrospun short fibrous sponges are demonstrated to possess good 3D adhesion onto chronic diabetic wounds in rats [7]. However, clinical applications for biomaterials remain hampered probably because of the “inertness” of synthetic ECM scaffolds [8,9]. Conversely, natural ECM contains useful structural and biochemical information, providing sufficient bioactive cues to trigger cell functions needed for tissue regeneration [10,11]. Natural ECM scaffolds are generated from decellularized ECM (dECM), either from decellularized cells (C-ECM) or decellularized tissue-specific ECM (TS-ECM) [12].

Considering the numerous advantages of dECM for cell growth and differentiation because of the retention of biochemical cues, dECM products have become an attractive platform for several bioengineering applications [13]. Nowadays, dECM applications in pioneering scaffold-manufacturing techniques such as 3D cell printing and electrospinning also bring the field closer to clinical translation. 3D cell printing, also known as bioprinting, enables the recapitulation of the unique features of human tissues and organs through the design of bioink and polymerization techniques [14,15]. Bioink is a formulation of cellular components and biomaterials [14,16]. These biomaterials could satisfy the requirements to print cell-laden constructs; however, tissue- and organ-specific dECM-based bioinks can recapitulate a cell-supportive microenvironment niche in 3D cell-printed constructs [16]. The use of the bioprinting method for printing of cell-laden structures can reportedly provide an optimized microenvironment for 3D-structured tissue growth [17]. Thus, the new paradigm of dECM-based bioinks has been deemed as a powerful modern technology. Recently, electrospinning has attracted notable attention as another scaffold manufacturing technique. Electrospinning is a high-throughput technique that fabricates high-porosity fibrous scaffolds with nano-/microsized ultrafine fibers, whose morphology and structure mimic those of natural ECM [13,18,19]. The retention of architecture in electrospinning is beneficial for cell growth and alignment, but the biomechanical components in dECM may play a great role in cell differentiation [13]. Moreover, dECM is often difficult to scale up to clinically desired shapes due to its physiochemical properties. Thus, the combination of dECM and electrospinning can reduce the limitations of dECM scaffolds and provide them with tunability.

Despite the broad use of ECM, its exact mechanisms for tissue repair remain elusive. This review discusses the characteristics and mechanisms of tissue- or cell-specific ECM, along with the preparation for 3D organoid models and preclinical applications of tissue repair. Furthermore, we address challenges in clinical application and future directions.

### Physiologic roles of TS-ECM in organ formation

ECM remodeling is crucial to organ formation and development. Among various organs, the intestine is an example of how ECM regulates normal organ morphogenesis [5]. In anurans tadpoles, the basement membrane of the tubular intestine thickens during intestinal metamorphosis. When induced by thyroid hormone, ECM proteins (including collagen, laminin, and fibronectin) increase, thereby inhibiting epithelial cell apoptosis in tadpoles [20]. Similarly, ECM remodeling is observed to play a central role in intestinal morphogenesis in rat [21] and mouse [22] models. Alternatively, other organs such as the lungs and the mammary and submandibular glands develop by epithelial branching. The branching process establishes the structure of these organs, and this process involves the repetitive formation of epithelial clefts and buds. The formation invades adjacent embryonic ECM, and the ECM composition and distribution shift over time. Thus, ECM remodeling provides structural integrity and regulates multiple cellular processes, such as cell growth, cell motility, and cell shape [23]. Meanwhile, the dysregulation of ECM components, structure, stiffness, and abundance may contribute to pathological conditions and exacerbate disease progression. For example, heavy scar formation is associated with abnormal ECM deposition [24], whereas osteoarthritis is linked to excessive ECM degradation [25].

### ECM composition

ECM displays a 3D macromolecular network providing both structural support and biomechanical signaling to mediate cell behaviors, such as adhesion, proliferation, migration, and differentiation [26–28]. ECM consists of collagens, fibronectin (FN), laminins, elastin, proteoglycans (PGs), glycosaminoglycans (GAGs), and several other glycoproteins [29].

In mammalian tissues, ECM is generally divided into two types based on location and composition: (1) the interstitial connective tissue matrix, which surrounds and supports most stromal cells, thereby providing structural scaffolding for tissues, such as skeletal, and smooth muscle tissues [5]; and (2) the basement membrane, which primarily supports the epithelium and separates it from the environmental stroma, such as tubular and hollow structure tissues [5,30]. Although the ratios of ECM composition and structure vary among different organs or tissues, common biomacromolecules have been extensively studied (Table 1). The most dominant and abundant protein within tissue ECM is collagen [31]. Specifically, collagen type I functions in forming fibrils, collagen type II is rich in cartilage, and collagen type IV serves as a constituent part of the basement membrane [32]. Collagen types I and II are the main components of ECM. FN is a ubiquitous ECM glycoprotein that plays a critical role in attaching onto cells through binding between ligands and receptors. Thus, FN can provide molecules within the ECM with adhesion sites, such as collagens, integrins, proteoglycan, and heparan sulfate [29]. Laminins also serve as adhesive sites for ECM biomacromolecules and receptors located on the cell surface [29]. Elastin fibers are large ECM structures that undergo repeated stretching forces and thus provide recoil to tissues [33]. GAGs are usually covalently bonded to proteins to form PGs, which are vital molecules in tissue development and homeostasis [34]. Hyaluronan (HA) is a linear form of GAG containing repetitive disaccharide units of N-acetyl-D-glucosamine and D-glucuronic acid. As a major constituent of the pericellular matrix of many cell types, HA attaches onto its cellular receptors or binds to its own synthases, thereby influencing various cell functions [35].

The composition of ECM is constantly updated. Matrix-bound nanovesicles, a subgroup of extracellular vesicles, have been recently found within ECM. They are embedded within it and have a tissue-specific microRNA cargo and membrane lipid structure that can play a significant role in the regulation of inflammation and healing processes [40].

### Role of ECM in inducing stem-cell fate

**Fig. 1:**
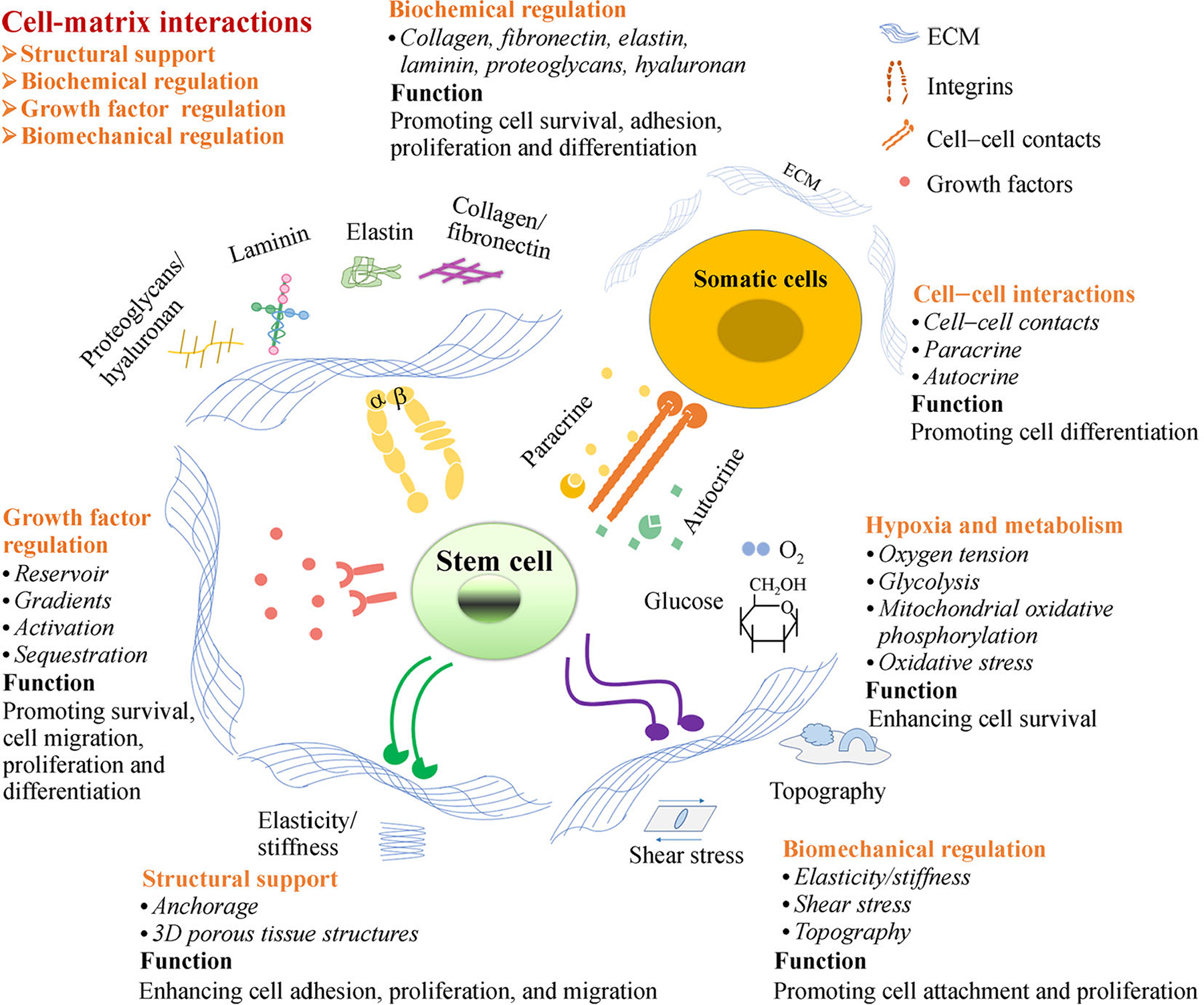
Role and composition of stem-cell niche. The stem-cell niches retain the stemness of adult stem cells in a quiescent state. When tissue is injured, the surrounding microenvironment actively signals stem cells to promote either self-renewal or differentiation to form new tissues. The niches include cell–matrix, cell–protein, protein–matrix, cell–cell interactions, hypoxia, and metabolism. Among these niche factors, cell–matrix interactions play a key role in prompting cell adhesion, migration, proliferation, and differentiation for tissue regeneration. The matrix regulates stem-cell behavior through structural supports, biochemical signaling, growth factor induction, and biomechanical regulation during tissue repair.

Accurately guiding stem cells to give rise to target cells is challenging due to the lack of defined inductors. As a natural niche, ECM provides a dynamic microenvironment for cell replication and differentiation when stem cells are activated [36]. The dynamic interaction in the microenvironment is also deemed as “dynamic reciprocity” [41]. With cell–ECM communication, ECM regulates stem-cell fate through structural support, biochemical composition, growth factors, and biomechanical factors [4] (Fig. 1) (Table 2).

First, ECM provides structural support for cells primarily because of the following: (1) the 3D structure of ECM allows an interconnected porous structure, and (2) the cross-linked fibrillar network and other large molecules provide rich cell-adhesion points [42]. Structural support is essential for cell adhesion, growth, and differentiation [43]. In 2020, Satyam *et al*. [44] reported a cell-derived ECM platform that could support podocyte proliferation, differentiation, and maintenance of the native phenotype.

With regard to biochemical composition, cells interact with the biochemical composition of ECM through transmembrane receptors. Integrins are the predominant transmembrane receptors on the surface of cells, connecting ECM proteins to the cytoskeleton within cells. They play crucial roles in various cellular activities, such as adhesion, proliferation, migration, differentiation, and homing [45–49]. Various integrin types are associated with the interactions between the cells and ECM, such as integrin α6β1, integrin α9, integrin β1, and integrin αvβ3 [48]. In 2020, Lu *et al*. [50] reported that integrin β1 knockout inhibits induced pluripotent stem cells (iPSCs)’ adhesion and migration across activated endothelial monolayers. In 2021, Han *et al*. [51] demonstrated that anti-human integrin β1 antibody could specifically target human iPSCs and differentiate into various lineages in a mouse model.

Furthermore, ECM proteins can bind and regulate growth-factor bioavailability, serving as a growth-factor reservoir. ECM proteins such as FN, collagens, and PGs alone or combined with heparin sulfate can connect to various growth factors, such as fibroblast growth factor (FGF), hepatocyte growth factor (HGF), and vascular endothelial growth factor (VEGF) [52]. Compared with unbounded growth factors, binding with ECM can potentiate their bioactivity. The phenomenon has already been observed in HGF, bone morphogenic protein (BMP)-2 and −4, acidic FGF, and insulin like growth factor (IGF)-1 [42,53]. ECM can also serve as microanatomic compartments. For example, due to the restrictions of basement membrane, asymmetric sequestration of bioactive factors occurs [52]. Thus, decellularized ECM having specific interactions with growth factors may generate dynamic and functional niches. In 2019, Ullah *et al*. [54] reported that replenishing human kidney ECM with VEGF results in more efficient differentiation of human iPSCs into endothelial cells (ECs).

Biomechanical factors including physical and mechanical forces can modulate the topography and microstructure of ECM in the local stem-cell microenvironment. Biomechanical factor changes can lead to variations in stem-cell shape and geometry. The microstructure of substrates could reportedly affect ECM protein binding [55,56]. Additionally, ECM stiffness has been identified as an important element in determining stem-cell fate in terms of lineage commitment [57,58] and self-renewal capacity [59]. For mesenchymal stem cells (MSCs), increased substrate stiffness enhances the osteogenic differentiation of MSCs [60,61], whereas soft matrix is inclined to induce chondrogenesis and adipogenesis [3]. ECM elasticity is another factor. In 2018, Hirata *et al*. [62] reported that the cardiac differentiation of iPSCs prefers highly elastic substrates *in vitro*. In 2020, Muncie *et al*. [63] demonstrated that substrates recapitulating embryo elasticity could promote human embryonic stem cells (ESCs) selforganization.

Recently, we have developed 3D human cell-based systems to replace the use of two-dimensional (2D) cell culture or animals for studying renal cytotoxicity [64]. To induce human urine-derived stem cells into renal tubular epithelial cells in 3D organoid culture, decellularized porcine kidney ECM is used as a culture supplement. Their results demonstrated that the levels of renal injury markers (CYP2E1 and KIM-1) in 3D organoids significantly increase in response to nephrotoxic agents (acetone and cisplatin). This 3D culture system with human stem cells and kidney-tissue ECM offers an alternative approach to renal-cytotoxicity testing [64].

## Preparation of dECM

Decellularization is a bioengineering technology used to isolate ECM scaffold from the cells inhabiting it. The ECM scaffold product possesses bioactive molecules from native tissue, which can be used for tissue regeneration and disease remodeling. The goal of ECM decellularization is to retain ECM compounds and structure and remove xenogeneic cell compounds, thereby avoiding immunoreaction. Thus, assessing changes quantitatively and qualitatively in ECM is critical. ECM can also be mediated by certain enzymes, which are responsible for ECM degradation after implantation *in vitro*, such as MMPs. Currently, commercially used ECM scaffolds are applied in wide-ranging bioengineering applications and are typically divided into C-ECM and TS-ECM (Table 3).

### Decellularization of cell-derived ECM

With various available treatments for decellularization, the careful monitoring of the combinations of physical, chemical, and enzymatic treatments is essential for the retention of the biochemical, biological, and biophysical properties of ECM [12]. Each of these methods may inflict damage to the structure and components of ECM, but no unified criteria exist for decellularization. Physical decellularization methods may be sufficiently harsh to alter ECM protein structures (e.g., collagen) and mechanical properties [67–70]. Chemical methods may break the connections between DNA and proteins, destroy the ultrastructure and growth factors, and denature ECM proteins [3,67,71–75]. Enzymes such as collagenase, lipase, trypsin, dispase, thermolysin, and nucleases [76,77] can remove cell residue or undesirable ECM components with high specificity. However, one limitation of enzymatic treatment is incomplete cell removal and impairment of recellularization [76]. Enzymatic treatments are insufficient for cell removal alone, so they are often combined with chemical detergents. Specific decellularization methods need to be optimized according to specific cell types, cell density, and ECM thickness [76]. Decellularization treatments are introduced systematically in the following section.

### Decellularization of tissue-specific ECM

In TS-ECM, many decellularization methods are designed to remove all cellular components [78,79]. The ideal procedure is to lyse cells and then wash away the cellular compounds from the tissue while retaining the ECM components and bioactive molecules. Thus, TS-ECM products retain natural ECM properties to form bioengineered tissues. After decellularization, the xenogeneic ECM scaffold could be recellularized with stem or progenitor cells, which differentiate into the original cell types in the tissue. Given their diverse applications for tissue regeneration, decellularization techniques must be tailored and integrated to meet the requirements for specific tissues. Decellularization methods that have been investigated include physical, chemical, and enzymatic treatments. Although some are commonly used, the optimal combination for decellularization depends on the tissue’s origin, characteristics, and intended use [76]. As for perfusion and immersion decellularization techniques applied to organs or tissues, they are applicable for tissues with extensive vasculature.

#### Physical treatments

The most common physical methods used for decellularization are to lyse or break the cell membrane or remove cells from the tissue matrix through temperature changes, mechanical force, and non-thermal irreversible electroporation (NTIRE). The mechanism involved in temperature methods is rapid freeze and thaw. After cell lysis, liquefied chemicals are used to treat the tissue. The purpose of this step is to degrade and wash out undesirable components. Temperature methods retain the ECM physical structure and are most suitable for strong and thick tissues. Mechanical-shaking force is commonly applied to organs with natural planes of dissection, such as the urinary bladder and the small intestine [80]. NTIRE is another alternative to lyse cells by using electrical pulses, which can disrupt the plasma membrane. However, NTIRE technology is suitable only for small tissues.

Interest in supercritical fluid technology to decellularize tissues is also growing. Supercritical carbon dioxide (scCO_2_) easily penetrates into biological tissues, thereby facilitating the removal of structural components of cellular membranes (lipids). The main advantages of this protocol are the significant reduction in processing time and the sterilizing effects. Nevertheless, the high pressure in a reactor can lead to the rupture of cells with subsequent removal of cellular fragments when the system is rapidly depressurized [81].

#### Prevalent chemical treatments

The appropriate chemical detergents are selected based on the tissue’s/organ’s thickness, ECM composition, and intended use. The prevalent chemical detergents used for decellularization include acids, bases, ionic detergents, and non-ionic detergents.

Acids and bases are used for solubilizing cellular cytoplasmic components and removing nucleic acids, including RNA and DNA. These chemicals can effectively disrupt both intracellular organelles, cell membranes, and some important molecules, including GAGs. Ionic detergents are used for effectively solubilizing plasma membranes and nuclear membranes by breaking protein–protein interactions [82]. Sodium dodecyl sulfate (SDS) is commonly used because it can effectively lyse cells while not damaging ECM significantly. Right after the cell membranes are lysed by SDS, the genetic contents are degraded by endonucleases and exonucleases. Non-ionic detergents disrupt lipid–lipid and lipid–protein interactions but leave protein–protein interactions intact. Triton X-100 is the most widely used non-ionic detergent [83].

#### Enzymatic treatments

Enzyme methods are used to destroy attachments between nucleic acid bonds. They interact with cells via adjacent proteins or other components of the cells. Collagenase, lipase, trypsin, dispase, thermolysin, and nuclease have been used to remove cells [76]. Serum has also been successfully used for decellularization due to the existence of nucleases [30].

Collagenase is appropriate for producing ECM scaffolds only when unbroken collagen structures are not required. Lipase is applicable when generating decellularized skin scaffolds. The function of lipase acids in the decellularization of skin dermis is degreasing and breaking the bonds among lipidized cells. Trypsin, a kind of serine protease, is also a common enzymatic agent for decellularization. Dispase is effective in separating undesired cells from ECM scaffold for its use in preventing cell aggregation. However, enzymes such as dispase and thermolysin are ineffective for removing cells inside tissues; they are more effective in combination with mechanical abrasion for complete cell removal [84]. Nucleases including DNase and RNase are often used for the cleavage of nucleic acids. Thus, nucleases are usually used to remove nucleic acids after cell lysis with physical pressure and chemical detergents [85].

Serum is commonly used in cell-culture systems because it contains many essential components that are beneficial for cell growth and propagation. The most extensively used serum is fetal bovine serum (FBS). Serum also contains serum nucleases, which can degrade the DNA and RNA remaining after cell lysis. Utilizing serum in decellularization methods has two extraordinary advantages: (1) retaining bioactive molecules in ECM compared with other reagents for decellularization [86]; and (2) degrading the DNA and RNA remaining after cell lysis, which can potentially induce immune responses [86–88].

In summary, the optimal decellularization approach is to minimize the loss of major bioactive matrix components and the xenogeneic immune responses simultaneously [30,80]. Single or combined decellularization methods are applied to achieve optimal efficiency according to the features of specific tissues and organs.

#### Handling of decellularized scaffolds

Decellularization yields multiple kinds of decellularized scaffolds, which can be further recellularized for *in vitro* and *in vivo* studies. Decellularized scaffolds are deemed the final products if the original ECM architecture is well retained [89]. Furthermore, decellularized C-ECM could be used in either its original format, or it can be fragmented, ground, or solubilized. Either 2D ECM sheets or complicated 3D structures comprising 3D scaffolds can be produced from these formats [12]. In other cases, post-processing techniques are needed to produce various products and thus meet research and clinical requirements, including the lyophilization, milling, and digestion of ECM, resulting in an injectable hydrogel [90]. It can be further cross-linked with genipin or glutaraldehyde to enhance the integrity [91].

## Applications of dECM

Considering the desired functions of ECM in mediating cellular behaviors, dECM is extensively used as a coating agent in 2D or 3D scaffolds [110]. Its utility in tissue regeneration and stem-cell lineage induction has now been widely examined among different tissues and organs. Based on the ECM source, we discuss the applications of C-ECM and TS-ECM separately.

### Cell-derived ECM

C-ECM is commonly used as a coating on biomaterial surfaces, but more sophisticated approaches exist. For example, the synthesis products of C-ECM can serve as 2D substrates for engineering tissues *de novo* or facilitating wound healing and regeneration [111]. According to different applications, C-ECM can be used as a biomaterial to regenerate tissues or promote cell-lineage commitment [111].

Compared with TS-ECM, an ideal scaffold material in tissue engineering, C-ECM is normally considered an *in vitro* niche, in which primary cells and MSCs can be rejuvenated to maintain their proliferation and differentiation capacity [112–114]. For instance, C-ECM has been demonstrated to refresh tissue-specific stem cells such as synovium-derived stem cells (SDSCs) [115–122], bone marrow-derived MSCs (BMSCs) [123–125], umbilical cord-derived MSCs (UCMSCs) [126,127], infrapatellar fat pad-derived stem cells (IPFSCs) [128–130], ESCs [131], periodontal ligament stem cells [132], and neural progenitor cells [133]. C-ECM also refreshes primary cells such as chondrocytes [134,135], nucleus pulposus cells [136,137], and hepatic cells [138] in proliferation and redifferentiation capacities (Table 4). This rejuvenation effect of C-ECM primarily occurs thorough anti-inflammation and antioxidation [121,122,126,135,139], which can reverse senescent stem cells and primary cells [127].

To explore the underlying mechanisms, adult human SDSCs are grown on C-ECM deposited by adult stem cells with varied chondrogenic capacity, including SDSCs (strong), adipose-derived stem cells (ADSCs; weak), and urine-derived stem cells (USCs; none), as well as C-ECM deposited by dermal fibroblasts (a non-stem-cell control) [119]. Despite the fact that expansion on C-ECM yields a large quantity of adult SDSCs with higher chondrogenic capacity than those on tissue-culture plastic (TCP), expansion on C-ECM deposited by SDSCs (with stronger chondrogenic capacity) yields SDSCs with less chondrogenic potential than those from other C-ECM groups. Intriguingly, SDSCs grown on C-ECM deposited by USCs display the highest expression of chondrogenic marker genes, aggrecan and type II collagen, which may be associated with the highest expression of basement membrane proteins. Furthermore, one basement membrane component, FN, has been evaluated in a recent study for its effect on the proliferation and differentiation capacity of stem cells by using CRISPR/CAS9-generated FN-knockout (FN1-KO) in human IPFSCs [129]. Wang *et al*. [129] found that FN1-KO promotes the proliferative capacity of human IPFSCs; however, this capacity is reversed during expansion on C-ECM generated by FN1-KO IPFSCs. The importance of FN in chondrogenic and adipogenic differentiation is also indicated in the FN1-KO IPFSCs and FN^–^ matrix microenvironment.

Another interesting study is to assess the influence of C-ECM expansion and immortalization on stem-cell proliferation and differentiation [130]. Wang *et al*. [130] found that human IPFSCs transduced with SV40 large T antigen (SV40LT) yields an increase in proliferation and adipogenic capacity but a decrease in chondrogenic potential. Interestingly, expansion on C-ECM generated by SV40LT transduced cells yields human IPFSCs with enhanced proliferation and chondrogenic potential but decreased adipogenic capacity. This outcome has been demonstrated to be highly relevant to the expression and distribution of basement membrane proteins.

### Tissue-specific ECM

Despite similar ECM composition among different tissues and organs, subtle differences in function, ratio, architecture, and stiffness of ECM can affect cellular interactions in determining cell fate [147]. Unlike C-ECM, which can refresh tissue-specific and non-tissue-specific stem/progenitor cells and primary cells, TS-ECM tends to function as a tissue-specific scaffold for stem/progenitor cells and primary cells in most cases [26]. Even without specific differentiation media, stem or progenitor cells still possess specific cell-lineage differentiation capacity based on particular interactions between cells and ECM [148]. Thus, compared with regular TCPs or natural scaffold such as collagens, TS-ECM is superior in maintaining [149] and guiding [150] stem-cell differentiation.

Depending on their application, TS-ECM products are generally divided by different organs (bone, articular cartilage, skeletal muscle, skin, and urinary bladder), different systems (musculoskeletal system, urinary system, and digestive system), or different germ layers (endoderm, mesoderm, and ectoderm). To address differences and its superiority to C-ECM, we classify TS-ECM products into four categories, namely, cell-culture supplements, cell sheets, tubular structures, and 3D structures according to different TS-ECM characteristics and applications (Table 5).

#### TS-ECM as supplements for in vitro 3D culture constructs

*In vitro* models aim to mimic the composition, ratio, and function of native tissues as closely as possible [151]. TS-ECM compounds could play a vital role in developing a proper *in vitro* cell-culture system. Compared with universal ECM such as collagen, TS-ECM can provide desirable cell–substrate interactions [147]. These interactions benefit cell proliferation and cellular functions, such as the differentiation capacity of stem or progenitor cells. Here, we focus on two post-processing products of TS-ECM for cell culture *in vitro*: powder and hydrogel. 3D matrix hydrogels often feature a soft, tissue-like stiffness and mimic the ECM that is naturally present in tissues. Using 3D ECM for cell-culture models presents several benefits as it enhances cell attachment and enables proper carrying of gases, nutrients, peptides, and proteins to the targeted cells, which promotes cell survival, proliferation, migration, and differentiation.

Tissue-like 3D cultures provide a promising tool to study the pathological changes to *in vitro* microenvironments. Pathogens such as viruses face varying conditions *in vivo*; however, suitable 3D tissue environments that impact pathogen spread need to be established. Recent studies [152] have developed tissue-like 3D cultures combining quantification of virus replication with imaging to study single-cell and cell-population dynamics. Investigators have analyzed human immunodeficiency virus-1 (HIV-1) spread between primary human CD4 T-lymphocytes using collagen as a tissue-like 3D model through computation technology. This study demonstrates that 3D environmental constructs restrict infection via cell-free virions but promote cell-associated HIV-1 transmission. Experimental validation identifies cell motility and density as essential determinants of the efficacy and mode of HIV-1 spread in 3D culture. 3D tissue constructs represent an adaptable method for the quantitative time-resolved analyses of HIV replication, spread, and interactions under *in vitro* 3D conditions [152].

The separation of ECM from tissues followed by decellularization and other processes (e.g., milling, pulverizing, lyophilizing, and freezing) are typical steps for producing ECM powder. TS-ECM powder derived from skin, muscle, and liver can be used as coating substrates for promoting targeted cell proliferation and maintaining the cell phenotype of the three cell types [147]. TS-ECM hydrogel is made using solubilized enzymatic procedures [153], which retain the full biochemical complexity of native tissue. Recent efforts have focused on recapitulating a wide variety of physiochemical cues of native ECM [154]. Our studies have demonstrated that synthetic skeletal muscle ECM (mECM) hydrogel, a combination of mECM, HA-based hydrogel, and heparin (HA-Hep), significantly improves the proliferation and differentiation of skeletal muscle precursor cells (MPCs) [30,87,88,155,156]. Additionally, TS-ECM from skin [155], liver [155,157,158], and kidney [64,159] efficiently induces tissue-specific stem cells to differentiate into dermal cells, hepatocytes, and renal cells, respectively, in 2D or 3D cultures.

TS-ECM based biomaterials in the bioengineering field have developed from simply coating cell-culture substrates to native ECM-mimicking scaffold design, aiming at recapitulating the exact dynamics, composition, and structure of native ECM [160]. Based on the different morphologies and topographical structures of TS-ECM, the applications can be further divided as cell-sheet tissue regeneration, tubular organ regeneration, and 3D tissue regeneration.

#### TS-ECM as cell sheet for tissue regeneration

**Fig. 2:**
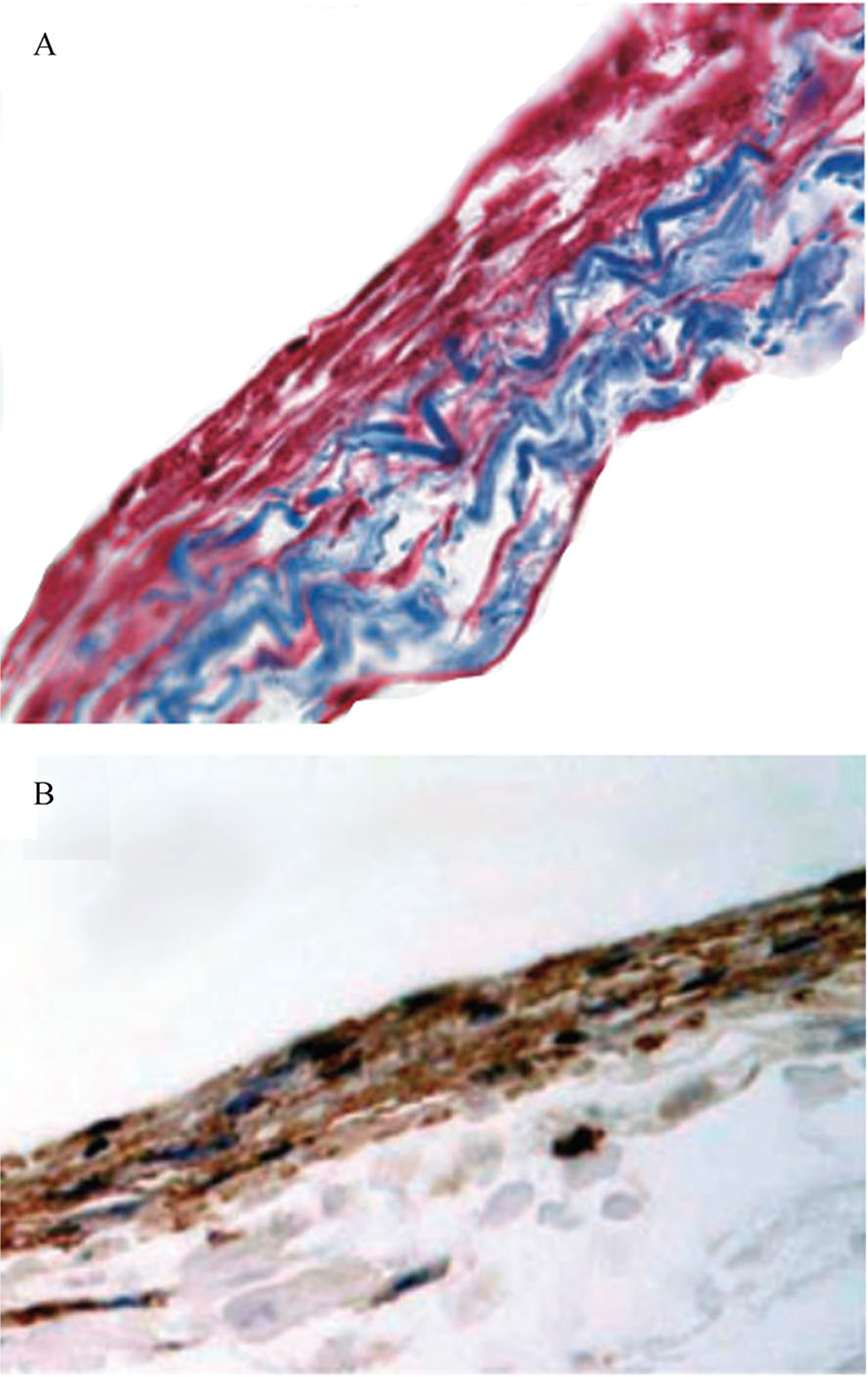
Cell-seeded decellularized small intestine submucosa scaffolds. (A) Masson trichrome staining of canine bone marrow stromal stem cells (red) seeded on SIS scaffolds (blue). (B) Immunohistochemistry staining of α-smooth muscle actin of bone marrow stromal cells (Brown). The photomicrograph of cell-seeded SIS scaffolds is adapted from *BJU International* [168] with permission.

Xenogeneic TS-ECM scaffolds, conveniently obtained using low-cost procedures, are typically fabricated as single-planar ECM sheets used for 2D tissue regeneration, such as skin (dermis) [161–163], cornea [164,165], and urethra mucosa [166,167]. Decellularized small intestinal submucosa (SIS) (Fig. 2) [168], bladder submucosa, and dermal matrix show promising results as inductive substrates for repairing full-thickness burns and postburn scar contractures [161,163,169]. Furthermore, decellularized porcine corneas using high hydrostatic pressurization show excellent optical properties without prompting an immune reaction when implanted into rabbit corneas [170].

#### TS-ECM for tubular organ regeneration

TS-ECM materials can be made into tubular scaffolds, which confer certain potential advantages, such as improved function or performance. Tubular TS-ECM can be used to regenerate blood vessels [171–178], esophagus [179–184], bladder [185–187], urethra [188,189], ureter [190], urinary conduit [191], bowel [192], and vagina [193].

SIS is one of the best established and most widely applied biomaterials [194]. Since it was reported for the first time in 1966 as a vascular substitute for replacing part of the aorta or vena cava in dog models [171–173], extensive research has been performed in the field. SIS-based scaffolds show good graft patency in small-diameter grafts [195]. However, they are observed to have a deficiency in forming intima, thickening media, and dilating grafts with large diameter [174,175]. Subsequently, decellularized vessels are demonstrated as another vascular scaffold. In 2000, acellular aorta scaffold seeded with human myofibroblasts and ECs showed great success following implantation in a rat model [176]. In 2008, the decellularization and recellularization of a whole heart was shown as a functional solid organ for the first time [196]. Large- and small-diameter vascular substitutes are produced from this process, after which the vascular tree could be recapitulated by relining vascular cells [177]. A recent study has reported that the integration of pericardial dECM and poly(propylene fumarate) has robust mechanical properties, adequate re-endothelialization, and tissuegrowth capacity *in vivo* [178].

Research on esophageal-tissue engineering has undergone rapid development in recent years. In 2000, Badylak *et al*. [179] successfully repaired esophageal defects in a dog model using acellular porcine SIS or urinary bladder submucosa. In 2011, Badylak *et al*. [180] first reported that xenogeneic ECM derived from porcine SIS promotes functional esophageal mucosa reconstruction for patients with endoscopic resection. In the same year, Clough *et al*. [181] reported that acellular porcine SIS matrix successfully repairs traumatic cervical esophageal perforation. In 2014, Syed *et al*. [182] reported that SIS could be consistently and reliably made into tubular scaffolds with good mechanical properties for esophageal-tissue engineering. In 2018, Luc *et al*. [183] reported a short biologic scaffold comprising decellularized esophageal matrix in a pig model, mimicking native esophagus in *in vitro* and *in vivo* characteristics. In 2019, a clinical-grade acellular matrix study reported an esophagus decellularization process, retaining native esophageal ECM structural, biochemical, and biomechanical properties without cytotoxicity, thereby meeting clinical-grade criteria and showing promise for clinical use [184].

**Fig. 3:**
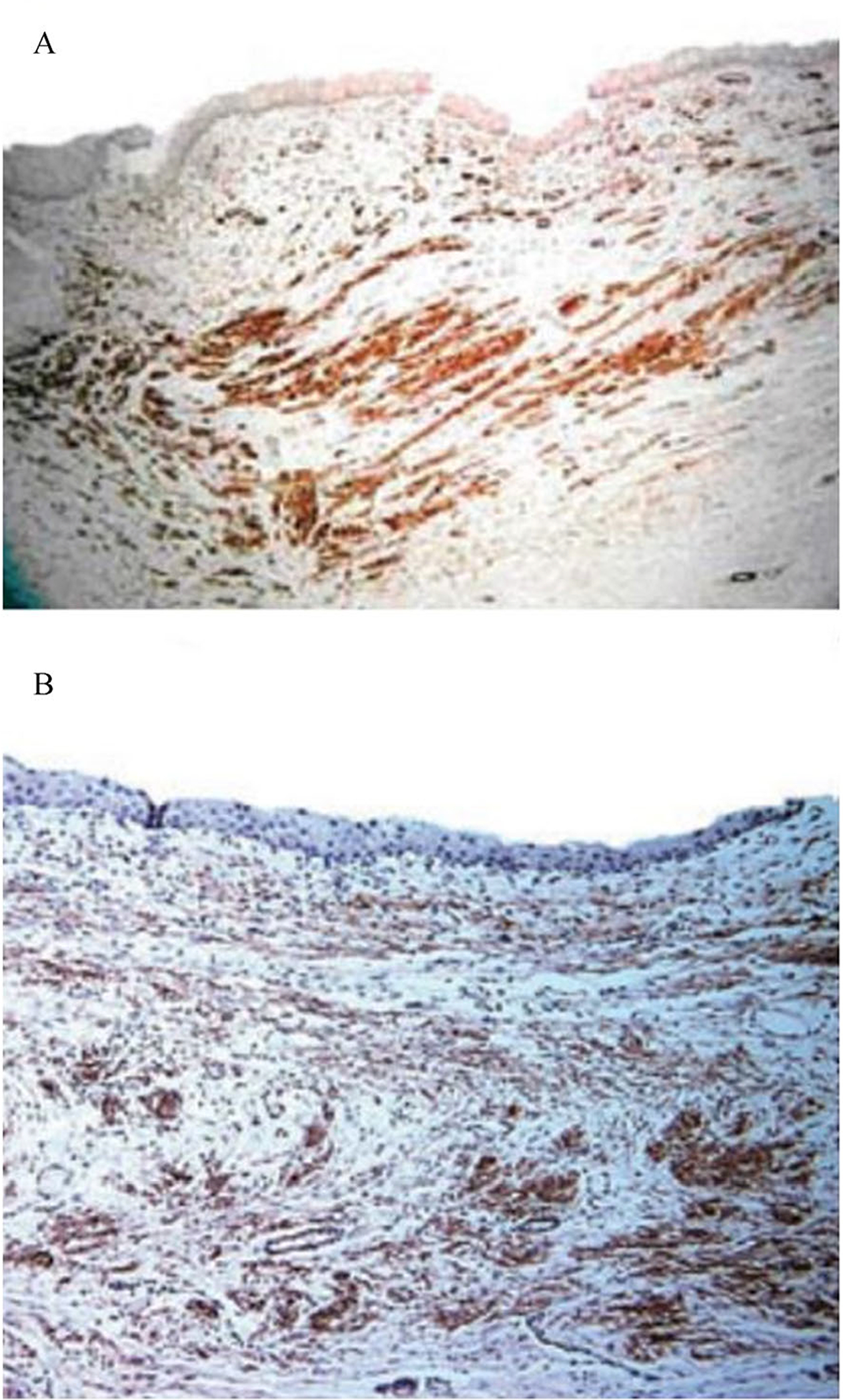
Bone marrow stromal cells-seeded decellularized extracellular matrix promoted *in vivo* bladder tissue regeneration. Both autologous bone marrow stromal cells-seeded (A) and bladder cells-seeded SIS scaffolds (B) expressed α-smooth muscle actin 10 weeks after transplantation in a canine model following partial cystectomy, assessed by immunohistochemistry staining. The images are adapted from *BJU International* [168] with permission.

Urinary-tissue regeneration is anatomically divided into urinary bladder, urethra, ureter, and urinary-conduit regeneration. Application of SIS for urinary-bladder reconstruction is extensively investigated. In 1995, Kropp *et al*. [186] reported that SIS could promote bladder regeneration in a rat model. In 2005, Zhang *et al*. [168] confirmed the result that SIS is a promising graft for regenerating the urinary bladder in a dog model (Fig. 3). Nowadays, natural porous polymer scaffolds are produced for bladder-bioengineering applications. In 2020, Zhang *et al*. [187] reported that SIS cross-linked with procyanidins could rapidly promote *in situ* tissue regrowth and regeneration of the bladder. As for urethral regeneration, since Kropp *et al*. [188] reported that SIS grafts for urethroplasty promote rabbit urethral regeneration in 1998, research on urethra regeneration has grown remarkably. To date, compared with synthetic scaffolds, tubular scaffolds derived from decellularized tissues can undergo subsequent remodeling with no inflammatory response *in vivo*. Matrix can be derived from SIS, dermal matrix, corpus spongiosum matrix (CSM), or bladder submucosa matrix (BSM). Among these matrices, acellular CSM and BSM seem to be the most appropriate scaffolds for urethra bioengineering because they possess molecular composition and mechanical and structural characteristics similar to those of native low urinary tract tissue [189]. Similarly, tubular scaffolds applied in ureteral regeneration are produced from decellularized native-tissue specimens such as SIS, amniotic membrane, ureter, blood vessels, or bladder tissue [190]. As for constructing artificial urinary conduits, the regeneration of the urinary conduit is studied primarily in animal models, and only one registered clinical trial has examined the clinical use of artificial urinary-conduit construction (unpublished data) [191].

Similar to urinary-conduit regeneration, research on bowel and vagina regeneration is also primarily performed in animals, such as rat [192] and porcine models [193]. However, graft shrinkage and scar-tissue formation are often observed after *in vivo* implantation. Apparently, keeping the lumen open with physical support is critical for tubular or hollow organ-tissue regeneration. For cell-seeded tissue, a promptly established blood network is required for the survival of implanted cells in the host [185]. Clearly, maintaining cell viability within ECM and preventing graft contraction after implantation require further investigation.

#### TS-ECM for multicellular-organism regeneration in vivo

Multicellular-organism regeneration requires a 3D framework to provide structural integrity and denote functional tissue boundaries, thereby delineating specific microenvironments [197]. Accordingly, the decellularization of whole tissues and organs provides scaffolds with tissue-specific 3D microarchitecture, serving as templates for whole-organ engineering [160]. The basic strategy for transplantable human-organ generation involves the venous perfusion decellularization of human or animal organs. The resulting product is a 3D framework with intact vasculature. Subsequently, the 3D scaffold is maintained in a bioreactor system to mimic the physiologic conditions of specific organs, such as electrical conduction, pressure gradients, pH, temperature, and oxygen concentration [198]. Next, the recellularization of 3D ECM scaffold proceeds by seeding appropriate cell types in a concentration that matches that for native cell distribution. The achievement of successful perfusion decellularization was first demonstrated on a whole rat heart in 2008 [198], followed by the liver, kidney, and lungs [199].

Several studies have reported the decellularization of liver tissue from animals [199–201]. The 3D ECM framework obtained from liver tissue has been proven to retain excellent functionality of multiple liver-cell types to grow *in vitro* [202,203]. In 2011, Baptista *et al*. decellularized a whole cadaveric liver organ by perfusing detergent through the native-liver vascular network, fabricating a natural ECM scaffold for liver regeneration *in vitro* [201]. In 2015, Mazza *et al*. [204] decellularized a whole human liver and successfully assessed *in vivo* quality and biocompatibility. Later, in 2017, Verstegen *et al*. [205] conducted a clinical series performing the decellularization process in whole liver. They generated a mild nondestructive decellularization protocol by using perfusion through the hepatic artery and the portal vein [205]. This protocol removes cellular DNA and RNA completely and is effective for generating constructs from whole human liver. These constructs contain ECM components, and the architecture of the liver is maintained. Above all, the utilization of artificial hepatic scaffold for liver bioengineering is gaining remarkable success. However, recellularization can be further improved using innovations of more desired bioreactors to better replicate native liver.

The goal of bioengineered lungs is to rehabilitate the architecture and functionality of the two seeding routes, the vasculature and the airway [199]. *In vivo* gas exchange is the primary outcome for evaluating the efficiency of artificial lungs. Initially in 2010, Petersen *et al*. [85] demonstrated the feasibility of recellularized artificial lungs based on a rat-transplantation model. In 2011, recellularized lungs transplanted orthotopically in rats partially restored respiratory function [206,207]. In porcine models in 2017, transplanted artificial lungs promoted gas exchange [208]. However, insufficient vascular barrier function and increased thrombogenicity resulted in graft failure [208]. Functional lung regeneration still has a long way to go even though remarkable achievements have been made. To build higher-level function, optimizing the recellularization and maturation of the grafts is necessary. Moreover, experiments based on large animal models need to be performed for preclinical trials before translation to human trials.

The two primary functions of kidneys are to maintain fluid balance and filter harmful substances, which are vital for human physiologic function. For patients with endstage renal diseases, kidney transplant is deemed the firstline treatment [209]. In the kidneys, various successful decellularization and recellularization strategies have been developed. For example, rat kidneys could produce dilute urine after recellularization and culture under perfusion [210]. However, although a piece of tissue like the structure of renal components is reconstructed *in vitro*, the function of renal tissue with a nephron structure has not yet been determined *in vivo* [211]. Moreover, the current techniques still have distinct limitations in precise cell arrangement, reconstruction of an entire vascular system, and a continuous urinary-collection system. These limitations impede obtaining complete and functional wholekidney organs. Additional studies need to be conducted prior to clinical applications.

#### Mechanisms for 3D tissue regeneration

Signaling pathways play crucial roles in substantial cellular functions (cell survival, self-renewal, attachment, proliferation, and differentiation) and tissue regeneration. Understanding the underlying signaling pathways is vital for 3D tissue regenerative repair. Key signaling pathways are involved in tissue regeneration in different systems (Table 6). These signaling pathways regulate stem-cell differentiation and 3D tissue regeneration in a complex cross-talk manner.

**Fig. 4:**
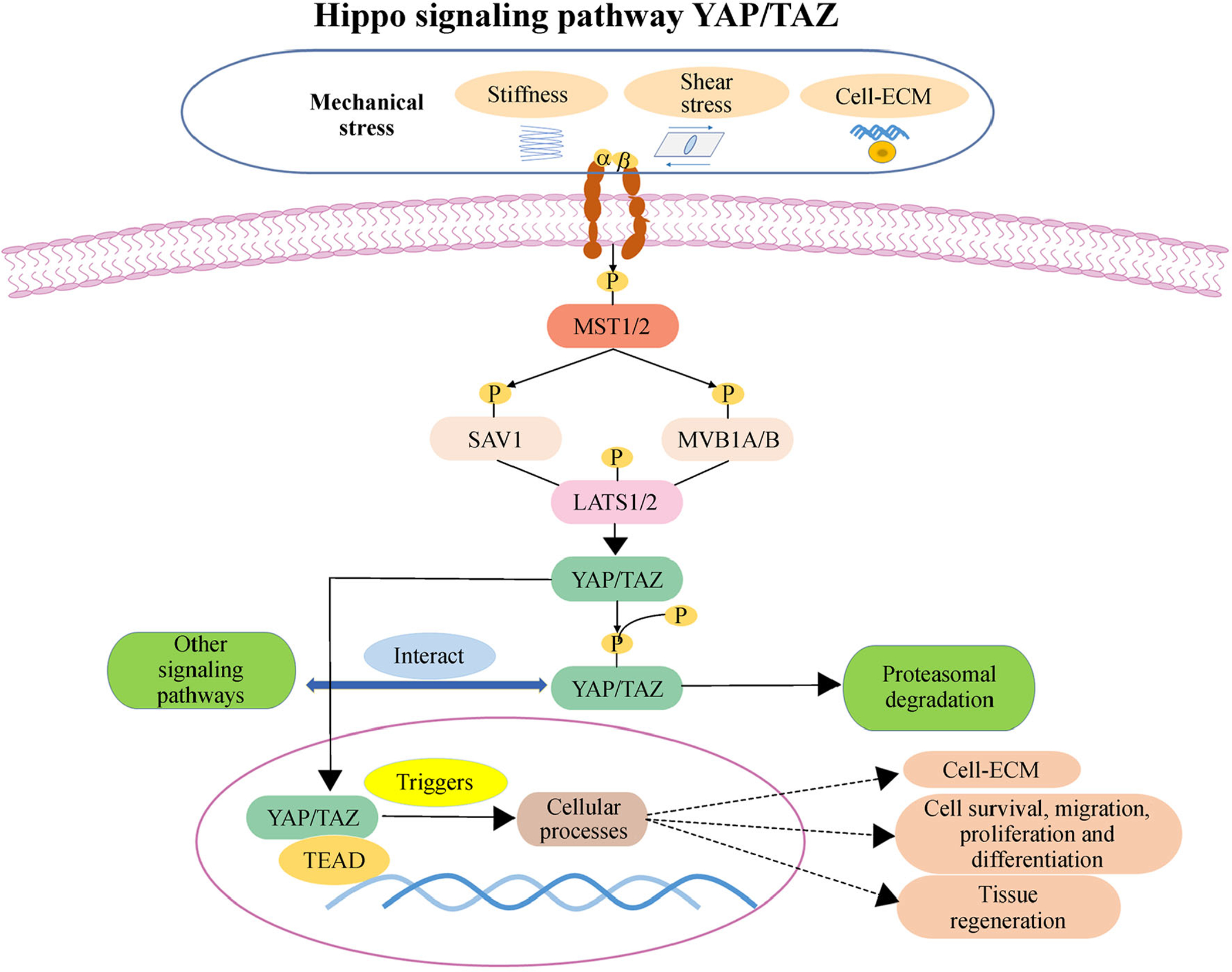
Hippo signaling pathway YAP/TAZ for regulating cell behaviors and tissue regeneration. The Hippo pathway is regulated by an intracellular network relaying a multitude of external inputs. Mechanical stress and cell-extracellular matrix (ECM) adhesion changes can regulate the Hippo pathway through integrin signaling. Activation of the Hippo pathway is associated with the phosphorylation of the core Hippo pathway kinases, including mammal Ste20-like kinase 1 (MST1) and MST2, Salvador 1 (SAV1), MOB1A and MOB1B, large tumor suppressor kinase 1 (LATS1) and LATS2, the transcriptional co-activators Yes-associated protein (YAP) and transcriptional co-activator with PDZ binding motif (TAZ), which leads to proteasomal degradation. Conversely, when the Hippo kinase cascade is not activated, unphosphorylated YAP/TAZ binding with TEAD transcription factor can activate specific genes, regulating ECM remodeling, cellular behaviors (cell attachment, proliferation, migration, and differentiation) and tissue regeneration.

Recently, the Hippo signaling pathway YAP/TAZ has been shown to play a pivotal role in regulating 3D tissue regeneration as a new signaling pathway [273]. The core of the Hippo pathway is defined as a serine/threonine kinase cascade, comprising mammal Ste20-like kinase 1 (MST1) and MST2, Salvador 1 (SAV1), MOB1A, and MOB1B, large tumor suppressor kinase 1 (LATS1) and LATS2, the transcriptional co-activators Yes-associated protein (YAP), and transcriptional co-activator with PDZ binding motif (TAZ) [274]. The Hippo pathway is regulated by external changes of stem-cell niche factors, such as mechanical stress and cell–ECM interaction [274]. The effects of these upstream signals are mediated by receptors embedded in the cytoplasm membrane, such as integrin complex (Fig. 4). After the cells sense the signals, the Hippo pathway is regulated by an intracellular network, rather than through dedicated receptors. Thus, following injury, the Hippo pathway can act as a universal pathway to regulate stem-cell behaviors for initiating tissue regeneration [273]. The Hippo pathway regulates stem-cell attachment, proliferation, self-renewal, and differentiation, such as ESCs [275], iPSCs [276,277], and MSCs [278], which are important for tissue regeneration. To date, it is reported to be involved in the regeneration of multiple organs, such as intestine [279], liver [280], skin [281], heart [241,282], and nervous system [283]. However, the downstream effects are closely associated with tumor development [284], thereby increasing the challenge in targeting the Hippo pathway for tissue regeneration.

## Challenges and future directions

Tissue-derived ECM is an elemental part of the body’s tissues, so it is critical to mimic its properties to develop 3D organoid models *in vitro* for drug screening, cell therapy, or disease modeling. Hydrogels such as collagen and matrigel are universal products extensively used as substrates for 3D cell cultures. However, the need for more special gels requires the development of various tissue gels. As the porosity, permeability, and mechanical characteristics of different gels vary, the natural origin of the ECM of specific tissues or organs needs to be recapitulated when these ECM gels are designed. TS-ECM compounds also need to be further characterized, controlled, and standardized to prevent variability in either C-ECM or TS-ECM.

For tissue repair in the body, ECM plays an important role in wound healing. As a complex physiologic reaction in response to trauma, would healing involves cellular and ECM events, biochemical reactions, growth factors, and cytokines. The goal for wound healing is scar-free restoration with less tissue shrinkage. Various possibilities have rendered ECM-based scaffolding technologies a turning point in regenerative medicine. To date, animal models have demonstrated that delayed collagen-deposition paired ECM remodeling is one of the traits for scarless wound healing [285]. However, some challenges exist for preclinical animal models, such as low reproducibility, ethical problems, and poor translation to humans. Moreover, the most prominent challenge is the inconsistency between healthy ECM scaffolds and the dysfunctional matrix that is the result of injuries. Dysfunctional matrix includes decreased or excessive ECM compounds [286], often accompanied with a change in soluble factors, such as transforming growth factor β [287] and cross-linking enzymes [288]. A proteomic study has also revealed that the composition of normal and pathological ECM exhibits a completely different profile [286]. Considering this finding, whether ECM scaffolds can provide the correct cues to regulate cell behaviors on pathological tissues is still unclear. To close the gap in knowledge, pathological ECM remodeling and genetically engineered ECM scaffolds offer two alternatives by improving the function and biocompatibility of ECM.

ECM remodeling is a healing process that offers promising therapeutic opportunities for many diseases [5]. Implanted ECM scaffold with a bioactive molecular and porous microstructure can enhance wound healing. For example, the immobilization of signaling molecules on the porous surface of scaffolds can promote cell proliferation, differentiation, and cell–matrix adhesion [289,290]. Selecting a specific enzyme to enhance tissue remodeling is important. One study has shown that curcumin treatment could accelerate wound healing by suppressing MMP-9 in a mouse model [291]. Moreover, attempts to genetically engineer ECM have achieved preliminary success in animal models. ECM sheets and hydrogels generated from porcine, which is alpha-gal deficient (with reduced immune rejection), show that 3D-generated transected anterior cruciate ligament can form in a goat model [292]. As TS-ECMs of different tissues share a common set of proteins, the role of individual ECM components in the unique functions of tissues and the healing process still needs further investigation. A robust and extensive proteomic analysis of TS-ECM components is critical to illustrate the tissue regeneration process induced by TS-ECM. In summary, a pro-regenerative matrix combined with the ECM remodeling of pathological tissues may bring us one step closer to scar-free tissue regeneration. TS-ECM in tissue repair could bring us closer to scarless wound healing.

In conclusion, mimicking the microenvironment of original tissues, TS-ECM and C-ECM possess remarkable promise for developing *in vitro* 3D culture systems and cell-based therapy. Tissue bioengineering in organoid constructions or 3D culture models offers a novel platform to study diseases and test new drugs. dECM products also provide therapeutic alternatives for the repair of injured or pathological tissues during tissue reconstruction. Compared with C-ECM, emerging evidence suggests that TS-ECM as a scaffold needs to be improved due to its unique biochemical, biological, and biophysical properties. This review highlights the physiologic roles of ECM in 3D organoid formation and tissue repair and presents the currently recognized applications of C-ECM and TS-ECM in modulating cellular construction development and organ-healing processes following tissue injury. To date, TS-ECM products have advanced to several formats such as powder, hydrogel, cell sheet, and decellularized tissue and organ for *in vitro* 3D structure culture models. Inevitably, tissue repair for wound healing will be refined in future applications.

The past few decades have witnessed substantial progress in TS-ECM or C-ECM developments. However, major hurdles remain in understanding the accurate and specific key ECM proteins and the ratio of these molecules for cell proliferation and targeted cell differentiation for 3D organoid culture and tissue repair. Thus, further basic research and preclinical testing are necessary before clinical translation.

## Figures and Tables

**Table 1:** Composition of ECM

ECM protein	Tissue sources	Functions
Collagen		Resists tensile and shearing forces, affects various cellular functions [29,36]
Collagen I (80%)	Skin, tendon, internal organs, organic parts of bone	
Collagen II	Cartilage	
Collagen III	Bone marrow, lymphoid tissues	
Collagen IV	Basement membrane	
Collagen V	Hair, surfaces of cells	
Fibronectin	Plasma, surfaces of cells	Cell adhesion sites, influences cellular behaviors [29,37]
Laminin	Basal lamina, placenta	Cell adhesion sites [29]
Elastin	Blood vessels, ligaments, skin, lung, bladder, elastic cartilage	Recoil [33]
Proteoglycans	Connective tissues, intracellular compartments, surfaces of cells	Resists compressive forces, provides recoil and participates in cell signaling and cellular behaviors [29,36]
Hyaluronan	Placenta, amniotic fluid, vitreous body, articular cartilage, dermis of skin	Lubricates, absorbs shock, affects cellular behaviors and signaling molecules [38,39]

**Table 2:** Role of ECM in inducing stem-cell fate

Role	Mechanism(s)	Function(s)
Structural support	Porosity, mechanical properties, cell–matrix communication	Regulating cell adhesion, growth, differentiation and forming 3D tissue structures [43]
Biochemical regulation	Integrins	Regulating cell proliferation, adhesion, migration, differentiation, homing [45,46,49,64]
Growth factor regulation	Reservoir, gradients, sequestration, activation, autocrine, paracrine	Regulating growth factor bioavailability dynamically [52]; maintaining stem-cell survival, self-renewal, differentiation [64–66]
Biomechanical regulation	ECM topography, microstructure, stiffness, elasticity	Modulating cell shape, tissue elongation, cell–ECM interactions; regulating stem-cell fate [55–57,59,62–64]

**Table 3:** Methodology of decellularized tissue or cell-derived ECM

Agents/techniques	Mode of action	Effects on ECM
Physical treatments		
Freeze and dry	Xenogeneic cellular compounds can be washed away after microscopic ice crystals disrupt cell membrane	Disrupt or fracture ECM fibers [92–94]
Mechanical-shaking force	Shaking action promotes cell debris removal from matrix	Disrupt ECM structure and clean up the cellular fragments [95–97]
NTIRE	Electrical pulse disrupts cellular membranes	Can disrupt ECM [98,99]
scCO_2_	Deeply penetrates into tissues and solubilizes non-polar molecules	Can disrupt ECM when the system is rapidly depressurized [81]
Chemical treatments		
Acids and bases	Disrupts both intracellular organelles and cell membranes	Break down collagen and GAGs and denature proteins or growth factors [95,100]
Ionic detergents	Solubilizes plasma membranes and nuclear membranes	Denature proteins via damaging bonds between proteins [82,101,102]
Non-ionic detergents	Disrupts bonds between lipids and between lipids and proteins	Beneficial to keep the ECM intact, may disrupt ultrastructure and GAGs [83,101,102]
Enzymatic treatments		
Trypsin	Cleaves cell adhesion from ECM	Extended exposure can destroy the structure of ECM, remove fibronectin, laminin, elastin, GAG [103–105]
Dispase	Cleaves collagen IV and fibronectin	Extended exposure can destroy the ultrastructure of ECM [95,106]
Nuclease (DNase and RNase)	Degrades nucleic acids	Hard to remove, may induce immune reaction [107–109]
FBS (serum containing DNase and RNase)	Retains bioactive proteins, degrades remaining DNA/RNA	Can minimize the loss of major bioactive proteins, decrease xenogeneic immune response [86–88]
Combined methodologies		
Shaking action + FBS	Optimizes approaches to remove xenogeneic cellular compounds by maintaining bioactive proteins and ECM structure	

ECM, extracellular matrix; GAGs, glycosaminoglycans; NTIRE, non-thermal irreversible electroporation; scCO_2_, supercritical carbon dioxide; FBS, fetal bovine serum.

**Table 4:** Applications of cell-derived ECM for *in vitro* tissue formation and *in vivo* tissue repairing

Application	ECM types	Cell types and animal models	Outcomes
Tissue regeneration			
Cartilage tissue	Porcine SDSCs	Porcine SDSCs *In vitro* and *in vivo* (13 minipigs)	Enhancing SDSCs’ expansion, chondrogenic potential, and repair of cartilage defects [139]
	Human adult vs. fetal SDSCs	Human adult SDSCs	Promoting adult SDSCs’ chondrogenic capacity by fetal ECM [140]
	Human fetal MSCs	Human adult MSCs	Promoting adult MSCs’ proliferation, multipotency, and stemness [141]
	Porcine chondrocytes vs. rabbit BMSCs	Rabbit chondrocytes	Supporting attachment and proliferation of chondrocytes [142]
	Porcine SDSCs	Porcine chondrocytes	Delaying chondrocyte dedifferentiation and enhanced redifferentiation [134]
	Porcine SDSCs vs. NPCs vs. SDSCs/NPCs	Porcine SDSCs	Guiding SDSCs’ differentiation toward the NP lineage [137]
	Porcine SDSCs	Porcine NPCs	Rejuvenating NPCs in proliferation and redifferentiation capacity [136]
Bone tissue	Mouse BMSCs	Mouse BMSCs *In vitro* and *in vivo* (nude mice)	Enhancing colony formation ability and retaining stemness [143]
	Human BMSCs	Human BMSCs *In vitro* and *in vivo* (nude mice)	Stimulating MSCs’ expansion and preserving their properties [144]
Nerve tissue	Rat Schwann cells	Rat dorsal root ganglion neurons	Improving axonal growth of dorsal root ganglion neurons [145]
Lineage commitment			
ESC differentiation	Murine ESCs line	Undifferentiated murine ESCs	Boosting early differentiation of ESCs [131]
Osteogenic differentiation	Rat osteoblasts	Human MSCs	Inducing osteogenic differentiation [146]
	Human BMSCs	Human BMSCs	Enhancing osteogenesis [124,125]
	Human BMSCs	Human BMSCs	Further enhancing proliferation and osteogenesis when combined with melatonin [123]
	Human USCs	Human BMSCs (passage 8)	Recharging BMSCs’ capacity in endochondral bone formation [125]
	Human UCMSCs	Human UCMSCs	Enhancing UCMSCs’ osteogenic differentiation by protecting from H_2_O_2_ induced senescence [127]
Chondrogenic differentiation	Rabbit articular chondrocytes	Human MSCs	Guiding chondrogenic differentiation [146]
	Porcine SDSCs	Porcine SDSCs	Promoting SDSCs’ proliferation and chondrogenic potential [115]
	Porcine	Porcine SDSCs	Maximizing SDSCs’ proliferation while maintaining chondrogenic potential when combined with FGF2 and low oxygen [116]
	Human fetal SDSCs	Human fetal SDSCs	Enhancing fetal SDSCs’ chondrogenic potential [118]
	Human adult vs. fetal SDSCs	Human fetal SDSCs	Enhancing SDSCs’ proliferation and chondrogenic capacity in a pellet culture under hypoxia [117]
	Passage 5 vs. 15 human IPFSCs	Passage 15 human IPFSCs	Promoting IPFSCs’ proliferation and chondrogenic potential by C-ECM deposited by passage 5 cells [130]
	Human adult SDSCs	Human adult SDSCs	Enhancing SDSCs’ chondrogenic potential compared with those in ECM [121]
	Porcine IPFSCs vs. SDSCs	Porcine IPFSCs	Enhancing IPFSCs’ proliferation and chondrogenic potential in both ECM groups [128]
Hepatic differentiation	Human liver progenitor HepaRG	Human DE cells	Aiding hepatic differentiation [138]

SDSC, synovium-derived stem cell; MSC, mesenchymal stem cell; BMSC, bone marrow-derived mesenchymal stem cell; NPC,nucleus pulposus cell; BM, bone marrow; ESC, embryonic stem cell; USC, urine-derived stem cell; UCMSC, umbilical cord-derived mesenchymal stem cell; IPFSC, infrapatellar fat pad-derived stem cell; DE, definitive endoderm.

**Table 5:** Applications of tissue-specific ECM in *in vitro* tissue construction or *in vivo* tissue regeneration

Application	ECM type	Seeded cell types	Culture condition(s)	Outcomes
*In vitro* 3D cultures				
Powder substrates	Acellular rat skeletal muscle ECM; acellular rat liver ECM; acellular swine skin ECM	Rat muscle cells; HepG2; human foreskin cells	*In vitro*	Promoting cell proliferation and differentiation [147]
Hydrogel substrates	Acellular skeletal muscle ECM combined with hyaluronan-based hydrogel and heparin	MPCs	*In vitro*	Promoting MPCs’ proliferation and differentiation [30]
Cell sheet tissue regeneration				
Skin (dermis)	Acellular human dermal ECM, allogeneic	None	*In vivo* (14 patients) [161]; *in vivo* (2 patients) [163]	Reducing scar and contracture [161,163]
Cornea	Acellular porcine cornea ECM, xenogeneic	None	*In vivo* (10 chinchilla bastard rabbits) [164]; *in vivo* (six eyes of rabbits) [165]	Biocompatible with the host’s epithelium [164,165]
Tubular organ regeneration				
Blood vessels	Acellular porcine aorta, xenogeneic	Human ECs and myofibroblasts	*In vivo* (5 Lewis rats)	Successfully implanted subcutaneously in a rat model [176]
	Acellular bovine pericardial ECM combined with poly propylene fumarate, xenogeneic	None	*In vitro* and *in vivo* (2 Lewis nude rats)	Remaining patent for two weeks in rat model [178]
Esophagus	Acellular porcine SIS, xenogeneic	None	*In vivo* (5 patients)	Promoting reconstruction of functional esophageal mucosa in patients [180]
	Acellular porcine SIS	Porcine BMSCs	*In vitro*	Meeting clinical-grade criteria, promising for clinical use [184]
Bladder	Acellular porcine SIS, xenogeneic	None, or seeded with dog UCs and SMCs	*In vitro* and *in vivo* (22 dogs)	Not achieving the desired bladder regeneration resulting in a subtotal cystectomy model as in the 40% cystectomy model [185]
	Acellular porcine SIS cross-linked with procyanidins, xenogeneic	None	*In vitro* and *in vivo* (48 New Zealand white rabbits)	Promoting *in situ* tissue regrowth and regeneration of rabbit bladder [187]
3D organ regeneration				
Liver	Acellular human liver ECM, allogeneic	hUVECs, hFLCs	*In vitro*	Decellularizing a whole liver organ for liver regeneration *in vitro* [201]
	Acellular human liver ECM, xenogeneic	LX2, Sk-Hep-1, HepG2	*In vitro* and *in vivo* (6 C57BL/6J mice)	Showing excellent viability, motility, proliferation and remodeling of the ECM in a mouse model [204]
Lung	Acellular adult rat lung ECM, allogeneic	Neonatal rat lung epithelial cells	*In vitro* and *in vivo* (344 rats)	Engineered lungs participated in gas exchange in a rat model [85]
	Acellular porcine lung ECM, xenogeneic	Human airway epithelial progenitor cells	*In vitro* and *in vivo* (3 pigs)	Demonstrating the feasibility of engineering of viable lung scaffolds in a porcine model [208]
Kidney	Perfusion decellularization of rat kidney and mounted in a whole-organ bioreactor, autologous	hUVECs, rat NKCs	*In vitro* and *in vivo* (68 Sprague-Dawley rats)	The resulting grafts produced rudimentary urine in an orthotopic transplantation model [210]

ECM, extracellular matrix; MPC, skeletal muscle precursor cell; SIS, small intestine submucosa; EC, endothelial cell; BMSC, bone marrow-derived mesenchymal stem cell; UC, urothelial cell; SMC, smooth muscle cell; HepG2, human hepatocarcinoma cell line; hUVEC, human umbilical vein endothelial cell; hFLC, human fetal liver cell; LX2, human cell line hepatic stellate cell; Sk-Hep-1, human cell line hepatocellular carcinoma; NKC, neonatal kidney cell.

**Table 6:** Mechanisms for 3D tissue regeneration

Function	Involved signaling pathway	Cell-matrix interaction related with genes and proteins
Musculoskeletal system		
Osteogenesis	BMP/TGFβ	Mesenchymal progenitors-BMP2-deficient mice [212], BMP4-deficient mice [213], BMP7-deficient mice [214]
	Wnt	Primary osteoprogenitors in Axin2^LacZ/LacZ^ mice-Wnt protein [215] Fracture callus tissues-PTH [216] Mesenchymal skeletal cells-peptide ligand with high affinity integrin (CRRETAWAC) [217]
	Notch	MSCs-Notch ligand (Jag1) [218–220]
Chondrogenesis	Wnt/β-catenin	Mesenchymal progenitors-ablation of β-catenin in mesenchymal condensations [221] Micromass of MSCs-protein kinase C inhibitor (PMA), p38 kinase inhibitor (SB203580) [222]
	TGFβ/Smad	FSTL1 KO MSCs-exogenous recombinant FSTL1 [223] Chondrocytes-Adamtsl2 KO growth plate [224]
	BMP	MSC pellets-BMP inhibitor (dorsomorphin) [225]
	BMP/TGFβ	hACs and hMSCs-BMP-2, TGFβ1 [226] SDSCs-BMP-2, TGFβ1 (dexamethasone absent) [227]
	IHH	Chondrocytes-PPR^−/−^ wild-type chimeric mice vs. Ihh^−/−^PPR^−/−^ wild-type chimeric mice [228] BMSCs-IHH, SHH [229]
Skeletal myogenesis	Wnt	Adult muscle stem cells-combining APC and β-catenin siRNAs [230] Satellite cells-Islr cKO mice [231]
	Wnt/IGF	Satellite cell-like reserve myoblasts-GSK-3 inhibitor (LiCl or SB216763), insulin [232]
	Notch	Adult muscle stem cells-COLV depleted mice (compound *Tg: Pax7-CreERT2; Col5a1*^*flox/flox*^*; R26*^*mTmG*^(*Col5a1* cKO)), CALCR ligand (Elcatonin) injection [233] Satellite cells-Syndecan-3 ablation [234]
Nervous system		
Neurogenesis in CNC	PI3K/AKT/mTOR	Cerebral organoids-mTOR activators (INSR, ITGB8, IFNAR1) and repressors (PTEN) [235]
	Notch	Neuronal progenitor cells-NOTCH2NL [236] hSpS spheroids-Notch inhibitor (DAPT) [237]
	Wnt/FGF	mESCs-FGF/Wnt agonist (CHIR)/RA [238]
	TGFβ/Shh/Wnt	Astrocytes-TGFβ, Shh, and Wnt activators [239]
Neurogenesis in PNS	c-Myc-TERT	Sensory axon-p53 inhibitor (PFTα), p53 activator (Tenovin-6) [240]
Circulatory system		
Cardiomyogenesis	Wnt	Cardiac organoids-Wnt agonist (CHIR) [241–243], WNT inhibitor (IWP2) [243]
	TGFβ	Cardiac organoids-TGFβ receptor inhibitor (e.g., SB431542) or overexpression of TGFβ receptor negative form [244,245]
	BMP	NKX2–5^+^CD31^+^ endocardial-like cells from hPSCs-BMP4, CHIR/BMP10, VEGF/BMP10 [246]
Angiogenesis	Notch	Vascular organoids-Notch inhibitor (DAPT), Notch ligands (Dll4, Notch3) [247]
	Wnt/VEGF-A	hPSCs aggregates-3D collagen I-matrigel gel driven by Wnt agonist (CHIR), BMP-4, VEGF-A, FGF-2 subsequently [248]
Digestive system		
Stomach tissue reconstruction	Wnt	Lgr5^+^ stem cells-matrigel containing Wnt activator (R-spondin1), Wnt3A [249]
		Axin2^+^/Lgr5^−^ stem cells-Wnt activator (R-spondin3) [250]
Intestine tissue reconstruction	Wnt	Lgr5^+^ ISCs-Wnt activator (R-spondin1), Wnt ligands [251–253]
	Wnt/Notch	Lgr5^+^ ISCs-Wnt inhibitor (IWP-2)/Lgr5^+^ ISCs-Notch inhibitor (DAPT) [254]
	Notch	ISCs-Notch ligands driven by transient Yap1 activation [255]
Hepatogenesis	Wnt	Lgr5^+^ stem cells-matrigel containing EGF, Wnt activator (R-spondin1) [256] Lgr5^+^ stem cells-HGF/Wnt activator (R-spondin1) [257]
	Hedgehog	Hepatocytes and ductular cells-Hh ligands [258] Stellate cells-JNK1 [259]
Urinary system		
Nephrogenesis	Wnt	Lgr5^+^ stem cells-Wnt receptor (Lgr5) [260] hPSCs-Wnt agonist (CHIR), Wnt inhibitor (DAPT) [261]
	Wnt, FGF	hPSCs-Wnt agonist (CHIR), FGF9 [262,263]
Urothelium regeneration	Hedgehog/Wnt	Stromal cells and epithelial cells in bladder-Shh-blocking antibody/stromal cells and epithelial cells-inactivation of essential component of Wnt pathway (Ctnnb1) [264]
	Hedgehog	Long-term bladder organoids-smoothened agonist (SAG), Hh inhibitor (vismodegib), genetic manipulation [265]
	Wnt/Notch	Urothelial organoids-Wnt agonist (CHIR)/urothelial organoids-Notch inhibitor (DBZ) [266]
Reproductive system		
Fallopian tube and oviduct tissue reconstruction	Wnt/Notch	Fallopian tube organoids-Wnt modulators (Wnt3a, R-spondin1, EGF, FGF10), TGFβ inhibitor (ALK4/5), BMP inhibitor (Noggin)/fallopian tube organoids-Notch inhibitor (DBZ) [267]
		Fallopian tube organoids-Wnt antagonist (PKF118–310)/fallopian tube organoids-Notch inhibitor (DBZ) [268]
Endometrium	Wnt	Endometrial organoids-Wnt activator (R-spondin1), Wnt inhibitor (IWP2), WNT3A, WNT7A, EGF, Noggin [269]
		Endometrial organoids-WNT3A, Wnt activator (R-spondin1), EGF, Noggin [270]
Vagina tissue reconstruction	Wnt	Vaginal organoids-EGF, TGFb/Alk inhibitor (A83-01), ROCK inhibitor (Y-27632), PALL Corporation (Ultraserum-G) [271]
Prostate tissue reconstruction	Notch	Prostate organoids-Notch inhibitor (DAPT) [272]

hAC, human articular chondrocyte; hMSC, human mesenchymal stem cell; SDSC, synovial-derived stem cell; IHH, Indian Hedgehog; PPR, PTH/PTHrP receptor; BMSC, bone marrow-derived mesenchymal stem cell; CNS, central nervous system; PNS, peripheral nervous system; hSpS, hindbrain/cervical spinal cord; mESC, mouse embryonic stem cell; ISC, intestinal stem cell;Hh, Hedgehog; hPSC, human pluripotent stem cell.
